# Case of Tetanus Treated by Bromide of Potassium

**Published:** 1869-09

**Authors:** Robert Brown

**Affiliations:** Carlisle


Case of Tetanus Treated by Bromide of Potassium.—By Robert Brown, Esq., Carlisle.—[The patient was a boy 12 years of age. About the end of September suppuration commenced around the root of the thumb nail, and terminated in the death and removal of the nail. On October 4th he fell asleep on the grass and awoke stiff and chilly. There was slight stiffness of the neck next day, and on October 8th there was rigidity of the spine and stiffness of the jaws.]

When admitted, two men carried him, perfectly rigid and bent, by the shoulders and lower extremities, from the cab to the ward.

State on admission.—He complains of stiffness in the back, neck and jaws; of pain in the neck, especially along the course of the sterno-mastoid muscles, and of great thirst. The sterno-mastoids, together with the muscles of the back and abdominal recti, are very tense. He can move his jaws apart about ¼ of an inch. During a paroxysm of pain and spasm, one of which occurred shortly after admission, he cried loudly, rested on his occiput and heels, and his countenance distinctly presented the "risus sardonicus." Pulse 112, irregular. Skin hot and dry. Bowels inactive. Ordered injection of warm water. To have milk, beef tea, arrow-root. Potass. bromid. gg. xx. every second hour.

13th October, vesp. Last night the bowels were freely moved; he slept little, but was quiet and easy. Has taken liquid nourishment freely. Paroxysms have been frequent and painful during the night; to-day they have occurred very often since 5 p. m. The boy perspires freely; can move his jaws apart fully ¼ of an inch. Pulse irregular, and so feeble that the beats can not be accurately counted. Urine normal in quantity and quality. Potass bromid. gr. xx. every hour.

15th, vesp. Patient during the early part of last two nights has suffered from severe paroxysms, occurring on an average four in the hour, and accompanied with a sense of choking, but after 1 a. m. he has become easier, and has slept a good deal. Since 5 p. m. to-day, paroxysms have occurred on an average 3 to 6 during the hour, though before that time he had been free from them, and had slept for three hours, being awakened however, to take his medicine, &c. Pulse 78, stronger, but still irregular. He perspires freely. Bowels acted after an injection for first time since night of admission.

16th. Patient slept well during the night till 3 a. m., when a paroxysm seized him, and almost threw him out of bed—such was its severity; it caused intense pain, and a wound of the tongue, by the sudden snapping together of his jaws. This morning he has taken milk and arrow root for his breakfast: lies quite easy, and perfectly free from pain, often dosing in sleep, with his mouth open fully an inch. He had to-day fifteen paroxysms in twelve hours. Tongue thickly coated with white fur. Breath fœted. Bowels inactive.

17th. Last night at 1.30 a. m., the house-surgeon was summoned by the nurse, and found the boy convulsed in a violent paroxysm. His arms were violently contracted, the sardonic grin well marked; opisthotonos extreme; breathing hurried and labored; blood and saliva streaming from the mouth; tongue bitten in two places; face livid. The paroxysm lasted for three minutes, and subsequently he lay for half-an-hour exhausted and prostrate. This morning he is easy, and sleeps hour after hour. Ordered injection. Hydrarg. subchlor., gr. ii.; pulv. rhei. gr. vi. M. et. ft. pulv.

18th. Patient had two violent and a few slight paroxysms during the night. Bowels acted twice. Pulse 84—89, weak. Ordered sherry ℥ss every fourth hour.

Vesp. During the day has had twenty-four paroxysms in twelve hours.

21st. Patient has passed easy nights, and slept well since last report Yesterday he slept nearly the whole day, being awakened every hour to take medicine. During the night there occurred fifteen paroxysms in four hours. To-day he thinks the stiffness in the neck is disappearing. Potass. brom. every second hour. Sherry ℥x daily.

24th, vesp. The boy has progressed rapidly and favorably since the 21st. Has slept a great deal, and prespired freely, odor of sweat being fœtid. To-day he has been restless, and has suffered from three paroxysms in six hours, though he was free from them in the morning. Pulse 86, regular. Ordered an injection of warm water, which brought away a large, dark, fœtid stool.

26th. Progress very satisfactory till 3 a. m. (this morning), when he became restless, and suffered from paroxysms frequently, on an average three an hour. This morning he looks haggard, and, during the spasmodic attacks, the sardonic grin, the opisthotonos, with the clenching of the jaws, and contractions of the arms, and pain in the neck are as well marked as on the 18th and 19th, when he took the paroxysms most frequently. Pulse feeble and irregular. Ordered whisky ℥ii. ℞. Potass. bromid. gr. xx. every hour. Ordered sherry, ℥ vi.

27th. Has become much easier, suffers less pain during a spasm, and sleeps hour after hour, save when he has to take his stimulants, fluid nutriment, or medicine which he does with avidity.

2 p. m. The house-surgeon was summoned by the boy's screams, and found him almost out of bed in a violent paroxysm—the nurse had left the ward for a moment. Ordered an enema.

1st November. Since the last paroxysm recorded, the boy has only had four, which occurred on the 29th. Improvement rapid. He sleeps well, and eats voraciously. Pulse 96, weak.

Vesp. To-day he has had three paroxysms, one of which took place after an ineffectual attempt to sit up in bed.

6th. Has had no paroxysm since last report, when he began to take the bromide every second hour. Pulse 90. Improves daily. He still wears the facial grin, and complains of pain along the course of the sterno-mastoids, especially when he moves his head. Bowels acted freely. ℞. Potass. brom. gr. xx. every third hour.

10th. The boy improves rapidly, and without relapse. For the last two days he has been out of bed, and sitting on a chair; if he attempts to walk he stumps along on his heels. Has still a broad grin, and stiffness in back and legs. His articulation is imperfect, owing to the rigid muscles. To discontinue whisky. Diet common; eggs ii. Sherry ℥ vi. Potass. bromid. gr. xx. ter in die.

25th. In a very satisfactory state.

2nd December. Discharged cured.—*Edinburgh Medical Journal, May* 1869, *p.* 993.

